# Association between tumor-stroma ratio and prognosis in solid tumor patients: a systematic review and meta-analysis

**DOI:** 10.18632/oncotarget.12135

**Published:** 2016-09-20

**Authors:** Jiayuan Wu, Caixia Liang, Manyu Chen, Wenmei Su

**Affiliations:** ^1^ Nutritional Department, The Affiliated Hospital of Guangdong Medical University, Zhanjiang 524001, China; ^2^ Department of Oncology, The Affiliated Hospital of Guangdong Medical University, Zhanjiang 524001, China

**Keywords:** tumor-stroma ratio, prognosis, clinicopathological features, solid tumors, meta-analysis

## Abstract

Tumor-related stroma plays an active role in tumor invasion and metastasis. The tumor–stroma ratio (TSR) in the pathologic specimen has drawn increasing attention from the field of predicting tumor prognosis. However, the prognostic value of TSR in solid tumors necessitates further elucidation. We conducted a meta-analysis on 14 studies with 4238 patients through a comprehensive electronic search on databases updated on May 2016 to explore the relationship between TSR and prognosis of solid tumors. The overall hazard ratio showed that rich stroma in tumor tissue was associated with poor overall survival (OS) (14 studies, 4238 patients) and disease-free survival (DFS) (9 studies, 2235 patients) of patients with solid tumors. The effect of low TSR on poor OS was observed among various cancer types, but not in the early stage of cervical caner. A significant relationship between low TSR and poor OS was also observed in the subgroup analyses based on study region, blinding status, and Newcastle–Ottawa Scale (NOS) score. Subgroup analyses indicated that cancer type, clinical stage, study region, blinding status, and NOS score did not affect the prognostic value of TSR for DFS. Moreover, low TSR was significantly correlated with the serious clinical stage, advanced depth of invasion, and positive lymph node metastasis. These findings indicate that a high proportion of stroma in cancer tissue is associated with poor clinical outcomes in cancer patients, and TSR may serve as an independent prognostic factor for solid tumors.

## INTRODUCTION

Tumor aggression is considered to be a multifactor process and is significantly influenced by the microenvironment. As the main component of a tumor microenvironment, the stromal compartment is essential for the maintenance of epithelial tissues and their malignant counterparts. Changes in the epithelium and the stroma frequently occur concurrently. The stroma could actually act as a barrier in tumorigenesis by restraining tumor cell proliferation in a normal tissue. However, cancer-related stromal components could actively facilitate the growth, differentiation, and movement of cancer cells in a tumor tissue [1]. In the “seed and soil” concept, cancer cells, called “seeds”, survive in a highly complex microenvironment of the surrounding stroma, called “soil” [2]. In fact, the stroma surrounding the cancer cells is not passive, as it plays an active role in supporting and nourishing tumor parenchyma [3]. The crosstalk between the neoplastic cells and the associated stroma contributes to the functional and structural support of the tumor microenvironment, leading to tumor progression and metastasis [4, 5]. Furthermore, aggressive tumor cells exploit the tumor microenvironment by residing in the stroma, transforming the surrounding tissue, and modifying the metabolism of the resident cells [6]. Thus, tumor-related stroma could provide novel and alternative strategies for biological intervention in the treatment of malignant tumors.

In the past decades, the main target for therapeutic interventions was solely based on the characteristics of the tumor cells. However, tumor-related stroma could also provide valuable information [7]. When tumor cells are targeted, the appearance of drug-resistant clones of the tumor cells caused failure in the therapeutic process because of the genetic instability of tumor cells, which are prone to antigen loss. Given the immutable and stable nature of stromal cells, they are less likely to exhibit mutation and drug-resistance, which could result in a stable curative effect and thus could be used to predict the prognosis and the therapeutic response of malignancy [8]. Therefore, the stromal compartment in the tumor tissue may contain more prognostic information than the tumor cells [9]. Tumor–stroma ratio (TSR) represents the percentage of the neoplastic cell component relative to the tumor-associated stroma in tumor tissue, and a low TSR implies a high (rich) proportion of stroma. Currently, the component of the tumor stroma in the pathologic specimen has attracted increasing attention as an important factor in tumor prognosis in cervical caner (CC) [10, 11], non small cell lung cancer (NSCLC) [12, 13], breast cancer (BC) [14, 15], and esophageal cancer (EC) [16, 17]. Although considerable advantages have been attained in this domain, the effect of TSR on predicting prognosis across different solid tumors remains uncertain. Several researchers reported that a stromal overgrowth in tumor tissue predicts unfavorable survival result. However, some researchers contradicted this finding. Considering that the identification of new prognostic factors is desirable for the effective determination of therapeutic strategies, we conducted a meta-analysis through quantitative evaluation to examine the prognostic role of TSR in patients with solid tumors.

## RESULTS

### Description of the included studies

**Figure 1:**
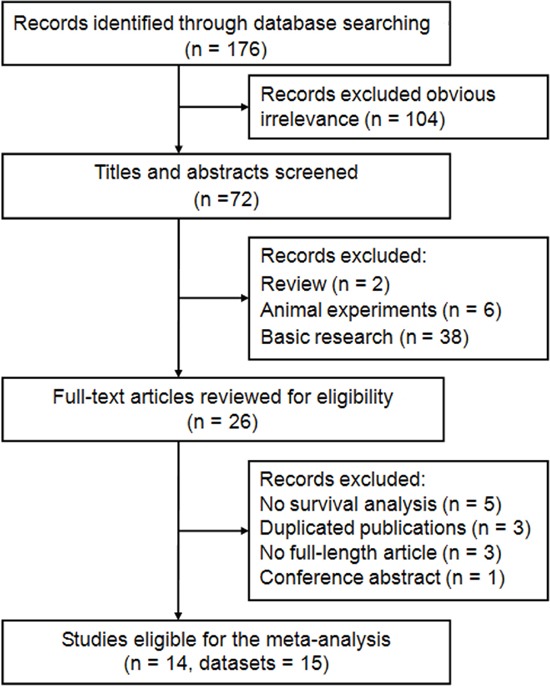
Flow diagram of the study selection process and specific reasons for exclusion in the meta-analysis 176 studies were preretrieved in accordance with the established search strategies. Of these articles, 104 were excluded because of clear lack of relevance. The remaining 72 studies were further screened out through browsing the titles and abstracts, and then 46 were removed based on the eligible criteria. After reading the full texts of 26 studies, 14 eligible studies were finally included in this meta-analysis.

The process of literature selection was shown in Figure 1. Initially, 176 papers were generated in the primary electronic search in major databases. According to the inclusion criteria, 14 eligible studies [10–23] with 15 datasets and published from 2007 to 2015 were included. The main characteristics of the included studies were listed in Table 1. A total of 4238 patients from China [10, 11, 13, 16, 18-20, 23], Thailand [12], and Europe [14, 15, 17, 21, 22] were diagnosed with various cancers, including CC [10, 11], NSCLC [12, 13], BC [14, 15], EC [16, 17], ovarian cancer [18], hepatocellular carcinoma (HCC) [19], colorectal cancer (CRC) [20–22], nasopharyngeal cancer [23]. Fourteen articles with 15 datasets reported the outcome of overall survival (OS) [10–23], and 9 studies with 9 datasets reported disease-free survival (DFS) [10-12, 14, 16, 17, 21-23]. Blinding status was reported in 13 studies, whereas 1 study with 2 datasets did not report the blinding status. All of the hazard ratio (HRs) and the corresponding 95% confidence interval (CIs) were obtained through multivariate analyses. The cutoff values in the included studies were all set to 50%. With regard to the language used, 12 studies were written in English, whereas the other 2 studies were in Chinese [13, 20]. According to the quality criteria, all cohort studies were of high quality and had scores of 6 or more. The quality assessments of all the published studies according to the Newcastle–Ottawa Scale (NOS) score are shown in Table S1.

**Table 1:** Main characteristics of the eligible studies

Study	Region	Cancer type	Clinical stage	Duration	Follow up (month)	Number	Blinding status	Cut off	Stroma-rich (%)	Analysis	Outcome	Language	Quality
Chen Y 2015 [18]	China	OC	I–IV	2001-2011	Until Dec 2014	838	Yes	≥ 50%	263 (31.4%)	Multivariate	OS	English	7
Pongsuvareeyakul T 2015 [10]	Thailand	CC	Ib–IIa	2003-2011	Median 73 (2-133)	131	Yes	≥ 50%	38 (29.0%)	Multivariate	OS, DFS	English	6
Lv Z 2015* [19]	China	HCC	I–IV	2003-2013	Until Aug 2014	300	NR	≥ 50%	75 (25.0%)	Multivariate	OS	English	6
Zhang TH 2015 [12]	China	NSCLC	I–IV	2007-2009	Median 51 (1-60)	404	Yes	≥ 50%	102 (25.2%)	Multivariate	OS, DFS	English	7
Zhang X 2015 [20]	China	CRC	II–III	2006-2010	Until Feb 2014	218	Yes	≥ 50%	58 (26.6%)	Multivariate	OS	Chinese	6
Liu J 2014 [11]	China	CC	Ia2–IIa	2005-2012	Until Jul 2013	184	Yes	≥ 50%	37 (21.0%)	Multivariate	OS, DFS	English	8
Zhang XL 2014 [23]	China	NPC	I–IVa	2004-2007	Until Dec 2012	93	Yes	≥ 50%	42 (45.2%)	Multivariate	OS, DFS	English	6
Dekker TJA 2013 [14]	Europe	BC	I–III	1986-1991	Median 41 (1-78)	403	Yes	≥ 50%	162 (40.2%)	Multivariate	OS, DFS	English	7
Huijbers A 2013 [21]	Europe	CRC	II–III	2002-2004	Median 58 (2-76)	710	Yes	≥ 50%	207 (29.2%)	Multivariate	OS, DFS	English	7
Wang ZF 2013 [13]	China	NSCLC	I–III	2000-2007	Until May 2012	73	Yes	≥ 50%	27 (37.0%)	Multivariate	OS	Chinese	6
Wang K 2012 [16]	China	EC	I–III	2007	Until Mar 2011	95	Yes	≥ 50%	30 (31.6%)	Multivariate	OS, DFS	English	6
de Kruijf EM 2011 [15]	Europe	BC	I–IV	1985-1994	Median 228 (168-288)	574	Yes	≥ 50%	388 (67.6%)	Multivariate	OS	English	7
Courrech Staal EF 2010 [17]	Europe	EC	I–IV	1990-2004	Median 23 (3-220)	93	Yes	≥ 50%	60 (64.5%)	Multivariate	OS, DFS	English	8
Mesker WE 2007 [22]	Europe	CRC	I–III	1980-2001	NR	122	Yes	≥ 50%	33 (27.0%)	Multivariate	OS, DFS	English	6

* There were two trials with two independent results in this article (sample size of 188 and 112, respectively).

NR none reported; OC ovarian cancer; CC cervical cancer; HCC hepatocellular carcinoma; NSCLC non small cell lung cancer; CRC colorectal cancer; NPC nasopharyngeal cancer; BC breast cancer; EC esophageal cancer; OS overall survival; DFS disease-free survival.

### Correlation between TSR and clinicopathological features

The relationship of TSR with clinicopathological features are illustrated in Table 2. A high proportion of stroma in tumor tissue was correlated with certain phenotypes of tumor aggressiveness, such as serious clinical stage (pooled odds ratio [OR] = 1.68; 95% CI = 1.20–2.51; *P* = 0.012; random effects), advanced depth of invasion (pooled OR = 1.56; 95% CI = 1.34–2.15; *P* = 0.006; random effects), and positive lymph node metastasis (pooled OR = 1.72; 95% CI = 1.16–2.55; *P* = 0.008; random effects). This finding indicated that a rich stroma in a tumor tissue may promote tumor invasion and aggressiveness. However, no association existed between TSR and certain factors, such as gender (pooled OR = 0.99; 95% CI = 0.75–1.30; *P* = 0.942; fixed effects), tumor size (pooled OR = 1.20; 95% CI = 0.93–1.56; *P* = 0.164; fixed effects), histological grade (pooled OR = 0.88; 95% CI = 0.68–1.14; *P* = 0.336; random effects), and lymphatic or venous invasion (pooled OR = 1.42; 95% CI = 0.87–2.31; *P* = 0.162; fixed effects).

**Table 2:** Meta-analysis of tumor-stroma ratio and clinicopathological features in solid tumors patients

Categories	Studies (no. of patients)	OR (95% CI)	*I^2^*(%)	*P*_h_	*Z*	*P*
Gender (male vs. female)	8 (1398)	0.99 (0.75-1.30)	29.9%	0.190	0.07	0.942
Tumor size (< 4cm vs. ≥4 cm)	6 (1307)	1.20 (0.93-1.56)	0.0%	0.819	1.39	0.164
Histological grade (moderate/well vs. poor)	11 (3201)	0.88 (0.68-1.14)^R^	50.2%	0.029	0.96	0.336
Clinical stage (I+II vs. III+IV)	9 (2236)	1.68 (1.20-2.51)^R^	70.1%	0.001	2.51	0.012
Depth of invasion (T_1_+T_2_ vs. T_3_+T_4_)	7 (1590)	1.56 (1.34-2.15)^R^	59.5%	0.022	2.73	0.006
Lymph node metastasis (negative vs. positive)	10 (2424)	1.72 (1.16-2.55)^R^	69.1%	0.001	2.67	0.008
Lymphatic or venous invasion (negative vs. positive)	3 (533)	1.42 (0.87-2.31)	47.0%	0.151	1.40	0.162

All pooled HRs were calculated from fixed-effect model except for cells marked with (random ^R^). *P*_h_ denotes *P* value for heterogeneity based on *Q* test; *P* denotes *P* value for statistical significance based on *Z* test.

### Correlation between TSR and OS

**Figure 2:**
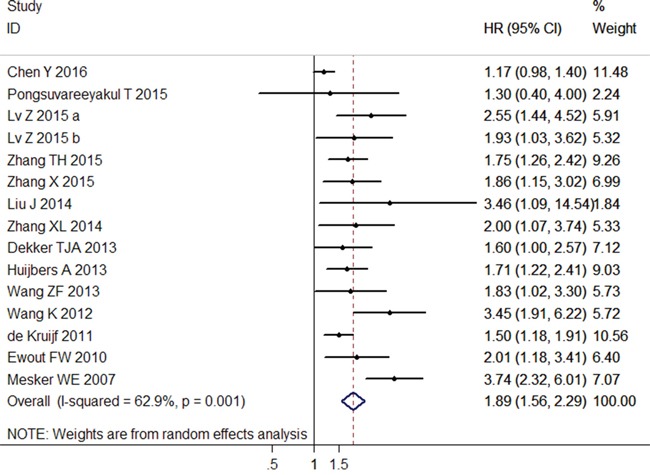
Forest plots of the overall outcome for overall survival (OS) Hazard ratios (HRs) for each trial are represented by the squares, and the horizontal lines crossing the square stand for the 95% confidence intervals (CIs). The diamonds represent the estimated pooled effect of the overall outcome for OS in all solid tumors. All *P* values are two-sided.

The combined analysis of 15 datasets from 14 studies showed that rich stroma in tumor tissue (low TSR) highly increased the risk of shortening the OS (pooled HR = 1.89; 95% CI = 1.56–2.29; *P* < 0.001; random effects) (Table 3; Figure 2). When the subgroup analysis was conducted by cancer type, the overall results revealed that low TSR significantly resulted in the poor OS of patients with CRC (pooled HR = 2.25; 95% CI = 1.40–3.61; *P* = 0.001; random effects), NSCLC (pooled HR = 1.77; 95% CI = 1.33–2.35; *P* < 0.001; fixed effects), HCC (pooled HR = 2.25; 95% CI = 1.47–3.43; *P* < 0.001; fixed effects), BC (pooled HR = 1.52; 95% CI = 1.23–1.88; *P* < 0.001; fixed effects), EC (pooled HR = 2.56; 95% CI = 1.72–3.79; *P* < 0.001; fixed effects), and other cancers (pooled HR = 1.22; 95% CI = 1.03–1.44; *P* = 0.022; random effects), but not with CC (pooled HR = 2.00; 95% CI = 0.85–4.74; *P* = 0.114; fixed effects) (Table 3). In the subgroup of the clinical stage, we observed that high TSR was still a favorable predictor of OS for Stages I–IV (pooled HR = 1.65; 95% CI = 1.33–2.04; *P* < 0.001; random effects), I–III (pooled HR = 2.48; 95% CI = 1.60–3.85; *P* < 0.001; random effects), and Stages II–III (pooled HR = 1.76; 95% CI = 1.33–2.32; *P* < 0.001; fixed effects), but not for Stages I–II (pooled HR = 2.00; 95% CI = 0.85–4.74; *P* = 0.114; fixed effects). Furthermore, this association did not only exist in the Eastern Asian population (pooled HR = 1.89; 95% CI = 1.45–2.45; *P* < 0.001; random effects), but also in the European population (pooled HR = 1.92; 95% CI = 1.43–2.60; *P* < 0.001; random effects) (Table 3). Moreover, the results did not change when the sample size, blinding status, and NOS score were included (Table 3).

**Table 3:** Pooled and subgroup analysis of main results for the meta-analysis of overall survival (OS)

Categories	Trials (Patients)	HR (95% CI)	*I*^2^ (%)	*P*_h_	*Z*	*P*
**OS**	15 (4238)	1.89 (1.56-2.29)^R^	62.9%	0.001	6.58	< 0.001
Cancer type						
CRC	3 (1050)	2.25 (1.40-3.61)^R^	72.5%	0.026	3.35	0.001
NSCLC	2 (477)	1.77 (1.33-2.35)	0.0%	0.891	3.91	< 0.001
HCC	2 (300)	2.25 (1.47-3.43)	0.0%	0.521	3.75	< 0.001
BC	2 (977)	1.52 (1.23-1.88)	0.0%	0.804	3.83	< 0.001
CC	2 (315)	2.00 (0.85-4.74)	18.6%	0.268	1.58	0.114
EC	2 (188)	2.56 (1.72-3.79)	44.4%	0.180	4.67	< 0.001
Others	2 (931)	1.22 (1.03-1.44)^R^	61.3%	0.108	2.29	0.022
Clinical stage						
I–IV	7 (2302)	1.65 (1.33-2.04)^R^	57.1%	0.030	4.53	< 0.001
I–III	4 (693)	2.48 (1.60-3.85)^R^	64.1%	0.039	4.04	< 0.001
II–III	2 (928)	1.76 (1.33-2.32)	0.0%	0.775	3.98	< 0.001
I–II	2 (315)	2.00 (0.85-4.74)	18.6%	0.268	1.58	0.114
Study region						
Eastern Asia	10 (2336)	1.89 (1.45-2.45)^R^	62.0%	0.005	4.77	< 0.001
Europe	5 (1902)	1.92 (1.43-2.60)^R^	65.9%	0.020	4.28	< 0.001
Blinding status *						
Yes	13 (3938)	1.86 (1.51-2.28)^R^	65.5%	0.001	5.93	< 0.001
NR	2 (300)	2.25 (1.47-3.43)	0.0%	0.521	3.75	< 0.001
Sample size						
≥ 100	11 (3884)	1.80 (1.45-2.23)^R^	65.9%	0.001	5.34	< 0.001
< 100	4 (354)	2.24 (1.67-2.99)	0.0%	0.423	5.43	< 0.001
NOS score						
≤ 6	8 (1032)	2.38 (1.94-2.93)	18.4%	0.285	8.23	< 0.001
> 6	7 (3206)	1.43 (1.28-1.60)	46.4%	0.082	6.14	< 0.001

* Blinding status represented that the evaluation of the tumor-troma ratio was blinded to the clinical outcomes.

OS overall survival, CRC colorectal cancer; NSCLC non small cell lung cancer; HCC hepatocellular carcinoma; BC breast cancer; CC cervival cancer; EC esophagus cancer; NOS Newcastle-Ottawa Scale.

We analyzed the heterogeneity of the included datasets based on the *P* value for heterogeneity. Table 3 illustrates that all of the included datasets of OS had extreme heterogeneity (*I*^2^ = 62.9%, *P*_h_ = 0.001). Thus, we used a random-effects model to estimate the overall HR for OS. When the subgroup analysis was conducted to assess the source of heterogeneity based on cancer type, clinical stage, study region, blinding status, sample size, and NOS score, the heterogeneity was obvious to be still significantly evident (Table 3).

### Correlation between TSR and DFS

**Figure 3:**
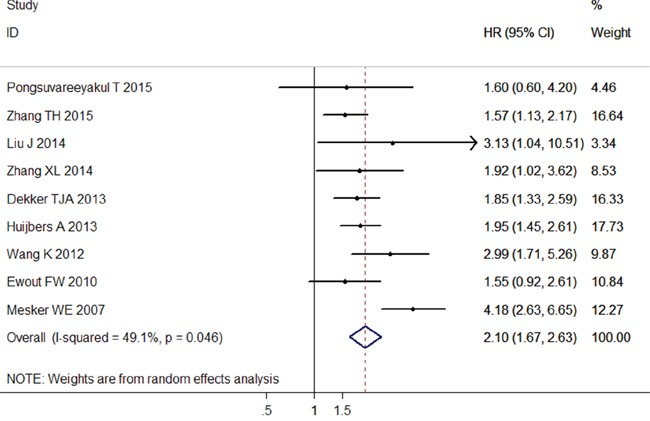
Forest plots of the overall outcome for disease-free survival (DFS) Hazard ratios (HRs) for each trial are represented by the squares, and the horizontal lines crossing the square stand for the 95% confidence intervals (CIs). The diamonds represent the estimated pooled effect of the overall outcome for DFS in all solid tumors. All *P* values are two-sided.

The data synthesis of the nine datasets showed a positive correlation between high ratio of stroma and worse DFS (pooled HR = 2.10; 95% CI = 1.67–2.63; *P* < 0.001; random effects) (Table 4; Figure 3), and this association was also significant in the subgroup analysis of CC (pooled HR = 2.11; 95% CI = 1.01–4.45; *P* = 0.049; fixed effects), CRC (pooled HR = 2.79; 95% CI = 1.32–5.89; *P* = 0.007; random effects), EC (pooled HR = 2.10; 95% CI = 1.43–3.08; *P* < 0.001; fixed effects), and other cancer types (pooled HR = 1.73; 95% CI = 1.39–2.15; *P* < 0.001; fixed effects) (Table 4). Similarly, the positive results were observed in the subgroup analysis based on clinical stage, study region, sample size, and NOS score (Table 4).

**Table 4:** Pooled and subgroup analysis of main results for the meta-analysis restricted to studies of disease-free survival (DFS)

Categories	Trials (Patients)	HR (95% CI)	*I*^2^ (%)	*P*_h_	*Z*	*P*
**DFS**	9 (2235)	2.10 (1.67-2.63)^R^	49.1%	0.046	6.41	< 0.001
Cancer type						
CC	2 (315)	2.11 (1.01-4.45)	0.0%	0.385	1.97	0.049
CRC	2 (832)	2.79 (1.32-5.89)^R^	86.5%	0.006	2.70	0.007
EC	2 (188)	2.10 (1.43-3.08)	64.6%	0.093	3.81	< 0.001
Others	3 (900)	1.73 (1.39-2.15)	0.0%	0.735	4.91	< 0.001
Clinical stage						
I–IV	3 (590)	1.62 (1.26-2.08)	0.0%	0.840	3.74	< 0.001
I–III	3 (620)	2.79 (1.65-4.70)^R^	75.6%	0.017	3.85	< 0.001
II–III	1 (710)	1.95 (1.45-2.61)	NA	NA	4.45	< 0.001
I–II	2 (315)	2.11 (1.01-4.45)	0.0%	0.385	1.97	0.049
Study region						
Easern Asia	5 (907)	1.88 (1.48-2.40)	14.3%	0.323	5.11	< 0.001
Europe	4 (1328)	2.18 (1.51-3.14)^R^	71.5%	0.014	4.19	< 0.001
Sample size						
≥100	6 (1954)	2.13 (1.58-2.87)^R^	61.1%	0.025	4.95	< 0.001
<100	3 (281)	2.05 (1.48-2.85)	30.4%	0.237	4.31	< 0.001
NOS score						
≤ 6	4 (441)	2.94 (2.18-3.95)	44.9%	0.142	7.12	< 0.001
> 6	5 (1794)	1.79 (1.51-2.12)	0.0%	0.703	6.69	< 0.001

DFS disease-free survival; NA not available; CC cervical cancer; CRC colorectal cancer; EC esophagus cancer; NOS Newcastle-Ottawa Scale.

All pooled HRs were calculated from fixed-effect model except for cells marked with (random ^R^). *P*_h_ denotes *P* value for heterogeneity based on *Q* test; *P* denotes *P* value for statistical significance based on *Z* test.

Similarly, a moderate heterogeneity was observed among the included studies of DFS (*I*^2^= 49.1%, *P*_h_ = 0.046). When subgroup analysis was performed, the heterogeneity was slightly reduced, but still statistically significant (Table 4).

### Sensitivity analysis and meta-regression analysis

**Figure 4:**
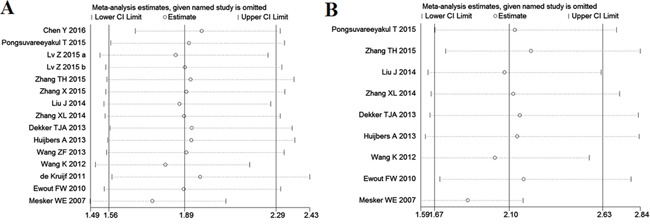
Effect of individual studies on pooled hazard ratios (HR) for the relationship between tumor-stroma ratio (TSR) and prognosis of solid tumors **A.** Sensitivity analysis for overall survival (OS). The vertical axis at 1.89 indicates the overall HR, and the two vertical axes at 1.56 and 2.29 indicate its 95% confidence interval (CI). Every hollow round indicates the pooled HR when the left study was omitted in a meta-analysis with a random model. The two ends of every broken line represent the respective 95% CI. **B.** Sensitivity analysis for disease-free survival (DFS). The vertical axis at 2.10 indicates the overall HR, and the two vertical axes at 1.67 and 2.63 indicate its 95% CI. Every hollow round indicates the pooled HR when the left study was omitted in a meta-analysis with a random model. The two ends of every broken line represent the respective 95% CI.

Sensitivity analysis suggested that no point estimate of the omitted individual dataset lay outside the 95% CI of the combined analysis based on the overall HR estimate of OS (Figure 4A) and DFS (Figure 4B). These results indicated that no individual study dominated the meta-analysis results, and the outcomes were stable and reliable.

We conducted meta-regression analysis to investigate the potential source of heterogeneity among studies for OS and PFS. The results showed that cancer type (*P* = 0.307), clinical stage (*P* = 0.829), study region (*P* = 0.172), blinding status (*P* = 0.764), sample size (*P* = 0.478) and NOS score (*P* = 0.079) did not contribute to the source of heterogeneity for OS. Moreover, the data demonstrated that cancer type (*P* = 0.685), clinical stage (*P* = 0.811), study region (*P* = 0.432), sample size (*P* = 0.489) and NOS score (*P* = 0.098) did not account for the source of heterogeneity for PFS.

### Publication bias

**Figure 5:**
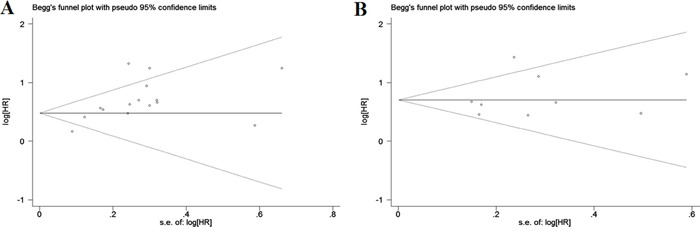
Begg's funnel plots for assessment of potential publication bias in studies of tumor-stroma ratio in patients with solid tumors Each study represented by one circle. The horizontal line represented the pooled effect estimate. **A.** Begg's funnel plot of publication bias for studies reporting overall survival (OS). **B.** Begg's funnel plot of publication bias for studies reporting disease-free survival (DFS).

No evidence of publication bias was found for the studies used for the meta-analysis for OS (Begg's test, *P* = 0.113; Egger' test, *P* = 0.106) and DFS (Begg's test, *P* = 0.466; Egger' test, *P* = 0.456). Moreover, the shape of the funnel plots did not show any obvious evidence of asymmetry for the outcomes of OS (Figure 5A) and DFS (Figure 5B). Hence, the results of the meta-analysis were robust and reliable.

## DISCUSSION

TSR, which is evaluated through the hematoxylin and eosin (H&E) stained sections, was first proposed by Mesker [22] in CRC patients, and has now been extended to other cancer types. The patients were divided into “stroma-rich” or “stroma-poor” groups according to the best cutoff of TSR = 50% because the prognostic difference between the two groups was most significant under this threshold. The quantitative approach of pathological morphology enables the accuracy and consistency of TSR evaluation relative to qualitative systems [9]. In contrast to methods with molecular markers, TSR detection is simple, rapid, and based on routine histological material without the need for additional special techniques and extra costs, thus facilitating repeated testing. Therefore, TSR is a convenient and useful tool for clinical application and facilitating the collection of prognostic information.

In this study, we conducted a meta-analysis on 4238 patients to evaluate the relationship between TSR and the prognosis of solid tumors, as the clinical implications of TSR remain unknown. From the statistical results, a high proportion of stroma to tumor parenchyma is significantly correlated to unfavorable prognosis (OS and DFS) in solid cancers. Thus, TSR could be an independent predictor of OS/DFS in patients with solid cancers. However, given that the biological characteristics of different tumors were varied, extra cautions is necessary when applying these results into clinical practice. We analyzed the combined HR through subgroup analysis on the basis of cancer types to further investigate the predictive value of TSR in various types of tumors. When the analysis was restricted to the survival outcome of DFS, a positive result was observed in all types of cancers, thus indicated that TSR could be a useful predictor for disease-specific mortality in patients with solid tumors. When the analysis was focused on OS, low TSR was observed to be significantly associated with worse survival in CRC, NSCLC, HCC, BC, EC, and other cancer types. Therefore, TSR can serve as a novel index for the prediction of all-cause mortality in solid tumors abovementioned. However, a borderline relationship was observed between TSR and poor OS in patients with CC. Similarly, the predictive value of TSR associated with the prognosis of solid tumors on an early stage (I–II stage) was determined to be insignificant, which also belonged to CC according to the subgroup analysis of the clinical stage. Thus, the prognostic role of TSR for early stage CC (I–II stage) appeared to be weakly supported by evidence. The consolidated results of Pongsuvareeyakul et al. [10] focused on cervical adenocarcinoma were contrary to the results of Liu et al. [11] with all of the subtypes of CC, indicating that the TSR has lower prognostic value among adenocarcinoma patients than in patients with other CC subtypes. These disparate outcomes could be partly explained by the difference among several histological types of CC, which leads to different effects of the standard prognostic variables [24, 25]. Given the small sample size and the limited number of selected papers, further studies are necessary to provide further insights into this topic.

Although sensitivity analysis supported the robustness of our results, the findings must be cautiously interpreted. In our meta-analysis, the heterogeneity of the OS and the DFS estimations were extreme, even when we conducted subgroup analyses. The marked heterogeneity could be probably attributed to the differences in the characteristics of the patients, cancer types, ethnicity, study protocol, and literature quality. Considering the confounding effect of such differences, we conducted a meta-regression analysis to further investigate the source of heterogeneity. However, none of the aforementioned confounding effects could completely explain the heterogeneity. Instead, we used a random-effects model to calculate the consolidated results to minimize the influence of heterogeneity to certain degree [26]. Moreover, as our meta-analysis was limited to published literatures, valuable information from studies with negative outcomes and small sample sizes could have been overlooked. Moreover, positive results with large populations were more likely to be published. Given the broad search criteria, no publication bias was observed among the studies concerning OS and DFS (*P* < 0.05), indicating that our results were stable and reliable.

We analyzed the correlation between TSR and the clinicopathological features that affected the survival outcomes of the cancer patients to further investigate the prognostic impact of TSR on solid tumors. According to our pooled results, the abnormal proportion of TSR was significantly associated with certain clinical parameters, such as clinical stage, depth of invasion, and lymph node metastasis. These results strongly supported the negative value of rich stroma on poor outcomes in solid tumors and tumor-related stroma plays a promoting role in tumor progression through different pathways. However, the mechanism underlying the promoting effect of stroma in solid tumors is still not fully understood.

During the early stage of tumor invasion, tumor cells penetrate the basement membrane and activate the stromal cells to form the tumor microenvironment [27]. Although none of the stromal cells are malignant, they interact with each other or with the cancer cells, thus leading to an abnormal phenotype and altered function because of the tumor microenvironment [28]. These changes further induce the recruitment of immune and endothelial cells, loss of cell adhesion, proteolysis, matrix remodeling, and cytoskeletal rearrangements, which are considered to be essential factors in the promotion of tumor growth and metastasis [29]. Overall, the formation of tumor-activated stroma results in the disruption of the epithelial tissue, immune evasion of malignant cells, and tumor invasion, which has been regarded as tumor stromatogenesis [30].

The components of tumor-related stroma are complex, including the extracellular matrix (ECM), various cell types, and different secreted factors. As an intermediary, the ECM assists the communication of cancer cells with stromal cells, such that cancer cells are able to colonize the microenvironment and form a metastasis [31]. Evidence has shown that abnormal expression of the factors that activate the ECM, such as secreted protein acidic and rich in cysteine [32], can promote tumor formation. Notably, the factors that degrade the ECM, including matrix metalloproteinases [33], also facilitate tumor initiation and invasion. In several cancer types, the activated fibroblast, also called cancer-associated fibroblast (CAF), is the predominant cell type within the tumor tissue rather than the cancer cells. Being different from the fibroblasts in a normal tissue, CAFs are not removed by apoptosis when the activating stimulus is attenuated. Thus, cancer has been metaphorically referred to as “a wound that never heals” [34]. During the early stages of tumor progression, CAFs play a role as suppressors of contact inhibition on cancer cells by enhancing the formation of gap junctions among the activated fibroblasts. During the later stage, CAFs function as promoters of tumor growth and progression after its activation by several tumor-secreted factors, such as fibroblast activation protein, α-smooth muscle actin, platelet-derived growth factor, basic fibroblast growth factor, and interleukin 6 [35, 36]. However, the exact cause of the transition of the CAFs from “tumor suppressors” to “tumor promoters” during tumor progression is still incompletely understood. Moreover, the promotion of CAFs on tumor progression has a broad range, inducing epithelial–mesenchymal transition of carcinoma cells, secretion of various growth factors, tumor metabolic reprogramming, preparation of metastatic niche, and therapy resistance [37]. In addition, arrays of growth factors and chemokines secreted by stromal cells, as well as cancer cells, into the stroma, such as the nuclear factor κB [38], transforming growth factor β [39], and tumor necrosis factor α [40], are chemoattractants for other non-cancer cells. These secretions facilitate the recruitment of non-cancer cells in the tumor stroma. The recruited cells include granulocytes, mast cells, monocytes/macrophages, fibroblasts, and endothelial cells, which are all necessary for tumor formation and extensively stimulate tumor progression [41]. Furthermore, the stromal cells can promote the infiltration and migration of tumor cells into the blood and lymph circulatory system; thus, tumor cells widely spread in the body. The stromal cells also promote metastasis by advancing angiogenesis and lymphangiogenesis, thus producing a significant negative effect on prognosis [42, 43].

Although significant progress has been attained in these current studies, certain limitations still persist in the clinical practice for TSR. First, the evaluation of TSR is mainly conducted postoperatively, and whether the TSR estimated from the preoperative biopsy specimens represents the entire tumor tissue is still uncertain. Although high consistency has been observed between the results of pre-operation and post-operation in a study of esophageal adenocarcinoma [44], the evidence is weak and thus cannot be extended to all tumors. Second, although TSR is suitable for the prediction of the prognosis of epithelial tumors, its prognostic role for other tumors is unclear. Furthermore, our meta-analysis has certain limitations. First, the number of relevant studies and the sample size were limited in the subgroup analyses based on cancer type, which make the consolidated outcomes unreliable. Thus, studies with large samples are necessary to determine a definitive value of TSR for the prognosis of different carcinomas. Second, the time of the follow-up period of each study was inconsistent, thus introducing heterogeneity to a certain extent. Third, although a subgroup analysis based on blinding status was conducted, the difference among the studies with similar protocols (experimental design, specimen preparation, and other relevant information) could have confounded the results. Fourth, clinical treatment is also a significant prognostic factor for cancer patients. Whether the effect of TSR is independent from clinical treatment is still unknown because several our included studies failed to control the latter. Fifth, although we did not impose limitations in language, only studies in English and Chinese were included in the meta-analysis. Finally, this is a meta-analysis at a study level. Therefore, confounding variables at the patient level were not incorporated into the analysis.

In summary, our present meta-analysis revealed that rich stroma in tumor tissue is associated with unfavorable prognosis, including OS and DFS, in patients with solid tumors. Given its convenience, quickness, and inexpensiveness in clinical application, TSR could be a useful tool for the prediction of prognosis and outcomes of solid tumors. However, further studies are recommended to explore the clinical importance of tumor-related stroma in tumor formation and development because all included studies in our meta-analysis are retrospective and the mechanism of stroma in tumor progression is still uncertain. In addition, the interactions between stromal components and tumors are critical for tumor aggressiveness and thus must be seriously considered for future novel therapeutic approaches.

## MATERIALS AND METHODS

### Search strategy and study selection

This meta-analysis was conducted according to the guidelines of the Preferred Reporting Items for Systematic Reviews and Meta-Analyses (PRISMA) [45]. The PubMed, Embase, Web of Science, Cochrane Library, and China National Knowledge Infrastructure databases were searched (last updated in May 2016) by using the following keywords: “tumor–stroma ratio or carcinoma–stroma ratio or cancer/carcinoma percentage (all fields), cancer or tumor or malignancy or neoplasm or carcinoma (all fields), and prognosis or prognostic or survival or outcome (all fields)”. The citation lists of the included studies were also screened for comprehensive search.

Publications were regarded as eligible when they satisfy all of the following criteria: (1) the cohort design reported the relationship between TSR and prognostic outcomes of solid tumors, such as OS, and DFS; (2) the patients with solid tumors were divided into two groups, namely, stroma-rich (low TSR or high carcinoma percentage) and stroma-poor (high TSR or low carcinoma percentage), regardless of the cutoff value; (3) the HRs for survival outcomes related to the TSR were provided in the original data or extracted from sufficient information; (4) the articles were written in any language as full papers; (5) articles with the largest patient cohort among duplicated publications by the same authors or institutes were included in the analysis; (6) the papers were not reviews, conference abstracts, editorials, or letters; and (7) the studies did not belong to basic research and animal experiments.

### Data extraction and quality assessment

Two authors (JYW and CXL) independently reviewed and extracted information from all of the eligible studies according to the criteria of study selection. Any disagreement between the reviewers was resolved by consensus. Data extracted from the studies included the first name of the authors, publication year, study region, cancer type, duration period, follow-up time, sample size, blinding status, cutoff value, clinical features, survival outcomes, HR estimation, and quality scores. Blinding status represented that the evaluation of the TSR was blinded to the clinical outcomes. In studies where the HRs and their corresponding 95% CIs of univariate and multivariate analyses were provided, only the latter was applied to the data synthesis because it is more precise and it considers the confounding factors. In the absence of results from multivariate analysis, HR was extracted from the univariate analysis or calculated using the Kaplan–Meier survival curves [46].

The quality of included studies was assessed by NOS according to the following categories: selection, comparability, and outcome of interest [47]. The total score of NOS ranged from 0 to 9, and we considered studies as high quality if they met at least six scores.

### Statistical analysis

The combined HR and 95% CI were used to assess the strength of TSR with survival endpoints (OS and DFS) based on the data extracted from the eligible studies. HR > 1 with 95% CI exceeding 1 indicated an increased risk of poor prognosis for patients with stroma-rich tumors. The statistical significance of the pooled HR was determined through Z–test. The results were considered statistically significant if *P* < 0.05. Subgroup analyses were conducted according to cancer type (at least two trials must report the same outcome for the same cancer type; otherwise, they will be assigned to a subgroup designated “Others”), clinical stage (“I–IV”, “I–III”, “II–III” and “I–II”), study region (“Eastern Asia” and “Europe”), blinding status (“yes” and “none reported”), sample size (“≥ 100” and “< 100”), and NOS score (“≤ 6” and “> 6”). Meta-regression analysis was also performed to determine the potential sources of heterogeneity. For the pooled analysis of the correlation between TSR and clinicopathological features (i. e., gender, tumor size, histological grade, clinical stage, depth of invasion, lymph node metastasis, and lymphatic or vascular invasion), the ORs and their corresponding 95% CI were combined to estimate the effect. STATA version 11.0 (STATA Corporation, College Station, TX, USA) was used for all statistical analysis. All statistical tests were two sided.

Heterogeneity assumption was qualitatively examined through the chi-squared test based on the Q statistic, and was considered statistically significant when *P*<0.05. Heterogeneity was also quantitatively estimated using the *I*^2^ metric, which is independent from the number of studies used in the meta-analysis (*I*^2^ < 25%, no heterogeneity; *I*^2^= 25% – 50%, moderate heterogeneity; *I*^2^ > 50%, extreme heterogeneity) [48]. We used the aforementioned qualitative and quantitative measurements to assess the between-study heterogeneity in this meta-analysis. When significant heterogeneity bad been observed among the studies (*P*<0.05 or *I*^2^ > 50%), the pooled HR estimation of each study was calculated using a random-effects model (DerSimonian and Laird method). Otherwise, a fixed-effects model was applied (Mantel–Haenszel method) [49]. Sensitivity analysis was conducted by sequentially omitting each individual study to validate the stability of the meta-analysis outcomes. The effect of potential publication bias on the outcomes was quantitatively evaluated through Begg's and Egger's asymmertry tests [50], and was visually evaluated using funnel plots. A two-tailed *P* value of less than 0.05 was defined as statistically significance.

## SUPPLEMENTARY MATERIALS TABLE


