# Supplementary material for: Association between tumor-stroma ratio and prognosis in solid tumor patients: a systematic review and meta-analysis
Source: Oncotarget. 2016 Sep 20;7(42):68954–65. doi: 10.18632/oncotarget.12135 (PMC5356603; doi:10.18632/oncotarget.12135)
Supplement: Supplementary file 1 [file oncotarget-07-68954-s001.pdf]

Association between tumor-stroma ratio and prognosis in solid tumor patients: a systematic review and meta-analysis

SUPPLEMENTARY TABLE

Supplementary Table 1: Quality assessments of all the eligible studies according to the Newcastle- Ottawa scale (NOS)

Author	Year	A	B	C	D	E	F	G	H	Total
Chen Y	2016	*	*	*	*	*	*	*		7
Pongsuvareeyakul	2015		*	*	*		*	*	*	6
Lv Z	2015			*	*	*	*	*	*	6
Zhang TH	2015	*	*	*	*	*	*		*	7
Zhang X	2015			*	*	*	*	*	*	6
Liu J	2014	*	*	*	*	*	*	*	*	8
Zhang XL	2014		*	*	*		*	*	*	6
Dekker TJA	2013	*	*	*	*	*	*		*	7
Huijbers A	2013	*	*	*	*	*	*		*	7
Wang ZF	2013			*	*	*	*	*	*	6
Wang K	2012		*	*	*		*	*	*	6
de Kruijf EM	2011	*	*	*	*	*	*	*		7
Ewout FW	2010	*	*	*	*	*	*	*	*	8
Mesker WE	2007	*	*	*	*		*		*	6

A: representativeness of the exposed cohort; B: selection of the nonexposed cohort; C: ascertainment of exposure; D: demonstration that outcome of interest was not present at start of study; E: comparability of cohorts on the basis of the design or analysis; F: assessment of outcome; G: follow-up long enough for outcomes to occur; H: adequacy of follow-up of cohorts.
